# Analysis of the Porphyrin Peak Shift and Fluorescence Lifetime in Gliomas with Different Tumor Grades, Intratumoral Regions, and Visible Fluorescence Status

**DOI:** 10.3390/diagnostics14232651

**Published:** 2024-11-25

**Authors:** Lisa Irina Körner, David Reichert, Marco Andreana, Angelika Unterhuber, Mikael T. Erkkilae, Jessica Makolli, Barbara Kiesel, Mario Mischkulnig, Thomas Rötzer-Pejrimovsky, Adelheid Wöhrer, Mitchel S. Berger, Rainer Leitgeb, Georg Widhalm

**Affiliations:** 1 Department of Neurosurgery, Medical University of Vienna, 1090 Vienna, Austria; lisa.koerner@meduniwien.ac.at (L.I.K.); jessica.makolli@meduniwien.ac.at (J.M.); barbara.kiesel@meduniwien.ac.at (B.K.); mario.mischkulnig@meduniwien.ac.at (M.M.); georg.widhalm@meduniwien.ac.at (G.W.); 2 Comprehensive Center for Clinical Neurosciences and Mental Health, Medical University of Vienna, 1090 Vienna, Austria; 3 Center for Medical Physics and Biomedical Engineering, Medical University of Vienna, 1090 Vienna, Austria; marco.andreana@meduniwien.ac.at (M.A.); angelika.unterhuber@meduniwien.ac.at (A.U.); mikael.erkkilae@web.de (M.T.E.); rainer.leitgeb@meduniwien.ac.at (R.L.); 4 Christian Doppler Laboratory OPTRAMED, Medical University of Vienna, 1090 Vienna, Austria; 5 Division of Neuropathology and Neurochemistry, Department of Neurology, Medical University of Vienna, 1090 Vienna, Austria; thomas.roetzer@meduniwien.ac.at (T.R.-P.); adelheid.woehrer@meduniwien.ac.at (A.W.); 6 Department of Neurological Surgery, University of California, San Francisco, CA 94143, USA; mitchel.berger@ucsf.edu

**Keywords:** protoporphyrin IX, 5-ALA, fluorescence lifetime, glioma, intraoperative visualization

## Abstract

**Background:** 5-aminolevulinic acid (5-ALA)-induced protoporphyrin IX (PpIX) fluorescence shows high sensitivity in detecting the tumor core of high-grade gliomas (HGG) but poor sensitivity for tissue of low-grade gliomas (LGG) and the margins of HGG. The characteristic emission peak for PpIX is known to be located at 635 nm. Recently, a second emission peak was described at 620 nm wavelength in LGG and the tumor infiltration zone of HGG. **Methods:** During surgery, samples from the tumor core and tumor infiltration zone of 43 WHO grade 2–4 gliomas were collected after preoperative 5-ALA administration, and their PpIX emission spectra, as well as fluorescence lifetimes, were determined by ex vivo analysis. Subsequently, the relative PpIX peak contribution (RPPC) was retrieved by calculating the integral of the two bands corresponding to the two emission peaks of PpIX (615–625 nm, 625–635 nm) and correlated with fluorescence lifetimes. **Results:** The mean RPPC decreased in samples with descending order of WHO grades, non-fluorescing samples, and infiltrative tumor regions, indicating a shift toward the 620 nm peak in porphyrin fluorescence. The porphyrin peak shift across all specimens correlated with lower fluorescence lifetimes (R: 0.854, R-squared: 0.729). **Conclusions:** The observed peak shift has important implications for fluorescence lifetime analyses since the lifetimes of other porphyrins contribute to the overall decay dynamics. Based on these initial data using fluorescence lifetime, this knowledge is of major importance, especially for detecting tissue from LGG that lack visible fluorescence, to further optimize the visualization of these tumor tissue using this promising imaging modality.

## 1. Introduction

A complete neurosurgical resection is crucial for a patient’s prognosis. Therefore, the development of novel techniques to support neurosurgical resection is of major importance. In recent decades, techniques such as neuronavigation, intraoperative magnet resonance imaging (MRI), ultrasound, and intraoperative neurophysiological monitoring have been widely used to improve patient outcomes. Additionally, fluorescence-guided surgery after oral administration of 5-aminolevulinic acid (5-ALA) showed promising results in increasing the rate of complete resections of the contrast-enhancing tumor and prolonged progression-free survival in high-grade glioma (HGG) patients [1,2,3]. 5-ALA is an endogenous metabolite precursor of the heme biosynthesis pathway and is converted by intracellular enzymes to the endogenous fluorophore that is the actual fluorescing compound protoporphyrin IX (PpIX) [4,5]. By using a modified neurosurgical microscope that is able to switch between white and blue excitation light, the presence of PpIX can be visualized during surgery. However, the results of visualizing tumor tissue in low-grade gliomas (LGG) or non-contrast-enhancing tumor margins in HGG with conventional 5-ALA fluorescence detection have demonstrated relatively poor sensitivity [6,7,8].

Based on the promising results in HGG, 5-ALA was also investigated in patients with other tumors, such as low-grade gliomas (LGG) [6,9,10,11,12,13]. However, 5-ALA showed poor sensitivity in visualizing pure LGG tissue as well as the tumor infiltration zone with only slight tumor cell infiltration in HGG using conventional fluorescence techniques [9,14,15,16]. Therefore, innovative quantitative methods to measure protoporphyrin IX (PpIX), the actual fluorescent compound of 5-ALA, were developed to improve the visualization of these usually non-fluorescing tumor tissues [17,18,19,20,21]. Recently, our group suggested frequency-domain fluorescence lifetime imaging (FD-FLIM) for the detection of even subvisual fluorescing areas in brain tumor surgery using a custom-built FD-FLIM system integrated into a neurosurgical microscope [22,23].

The common assumption in the literature represents an emission spectrum of PpIX located at 635 nm wavelength [24]. However, PpIX has a second peak at 620 nm aside from the well-known peak at 635 nm wavelength [25,26]. Recent studies reported that due to the multiple photochemical states of PpIX, both emission peaks should be taken into account when evaluating PpIX fluorescence in LGG as well as HGG tissue [25,26]. Moreover, the first observations found a shift of the PpIX emission spectrum toward the peak at 620 nm in LGG tissue as well as the tumor infiltration zone of HGG [26]. In an independent study, we found an additional shifted peak at 627 nm aside from the characteristic main PpIX peak at 635 nm that was suspected to be present, especially in tissues with very low PpIX concentrations [27]. The implications of this peak shift for intraoperative detection of low infiltrative glioma tissue thus require further investigation. Interestingly, a recent retrospective analysis found that not only the second photo-state of PpIX but also other porphyrins, such as coproporphyrin III, highly contribute to the peak at 620 nm, adding to the spectral complexity of the autofluorescence background [28].

Based on initial findings in the literature and our observations [25,26,27], we designed this study to investigate the emission spectra of samples collected from the tumor core and the tumor infiltration zone during surgery of gliomas with different CNS WHO grades (grades 2–4) after preoperative 5-ALA administration and correlated these data for the first time with FD-FLIM measurements. Furthermore, we compared these data with glioma samples without prior 5-ALA administration.

## 2. Materials and Methods

In this study, we included (*n* = 43) adult patients (≥18 years) who underwent 5-ALA fluorescence-guided resection of a suspected diffusely infiltrating glioma WHO grades 2–4 at the Department of Neurosurgery, Medical University Vienna, and in whom tissue samples for ex vivo measurements with FD-FLIM were available [22]. Patients had to be excluded from this study if their final histopathological diagnosis was different from diffusely infiltrating glioma WHO grades 2–4. This study was conducted according to the approved IRB protocol—Medical University of Vienna 419/2008, amendment—Ethics Commission Medical University Vienna—and patients gave informed consent.

### 2.1. Preoperative Course

In all patients, a preoperative diagnostic magnetic resonance imaging (MRI) study was performed usually within one week before surgery, including contrast-enhanced T1-weighted images for neuronavigation. Depending on the tumor localization, functional and/or diffusion tensor imaging MRI was obtained. According to preoperative MRI, tumors were classified into gliomas with significant and non-significant contrast enhancement (CE). Tumors with significant CE showed a typical “ringlike” pattern of CE in T1-weighted images. In contrast, tumors with non-significant CE showed “no CE”, patchy/faint” (unspecific) CE, or “focal” (small regional) CE. In patients who received 5-ALA before surgery, an oral dosage of 20 mg/kg body weight was administered approximately 3 h before anesthesia.

### 2.2. Neurosurgical Resection and Tissue Collection

All tumor resections were performed using neuronavigation guidance and a specified neurosurgical microscope (KINEVO; Carl Zeiss Surgical GmbH, Oberkochen, Germany) with the ability to switch between conventional white light and blue excitation light. During surgery of gliomas presenting with significant contrast enhancement on MRI, tissue samples were collected from the contrast-enhancing tumor area and the region outside the enhancing area if safely possible. In cases of gliomas without significant contrast enhancement on MRI, tissue samples were collected from regions within the T2/FLAIR hyperintensity. After 5-ALA administration, each collected tissue sample was screened for visible 5-ALA fluorescence status by the performing neurosurgeon during surgery and documented as fluorescence positive or negative. According to our clinical routine, all patients were protected from strong light sources for at least 24 h after surgery to avoid phototoxic reactions after oral 5-ALA administration. In detail, after surgery, patients and staff were obliged to lower the window blinds in their rooms, avoid strong light sources as well as avoid walks outside the hospital building for at least 24 h. Additionally, we included non-tumorous tissue samples derived from patients who underwent a neurosurgical procedure different from glioma surgery. These samples were collected whenever it was safely possible in cases requiring the removal of non-tumorous tissue during the neurosurgical approach.

### 2.3. Tissue Preparation

Within one hour after tissue collection, all collected tissue samples were immersed in artificial cerebrospinal fluid (CSF) at room temperature and protected from light for safe transportation to the Department of Physics, MUV, in order to perform FD-FLIM analysis. The artificial CSF (NaCl 0.9%, B.Braun©, Maria Enzersdorf, Austria) contains isotonic components, including sodium chloride, calcium chloride, magnesium chloride, potassium chloride, and glucose, to preserve the viability of the tissue samples. Depending on the size of each tissue sample, multiple regions of interest were examined using FD-FLIM. After the FD-FLIM analysis was completed, all tissue samples were directly transferred to the Neuropathology Department for routine histopathological work-up.

### 2.4. Imaging System and Post-Processing

FD-FLIM and spectroscopic measurements were acquired with a multimodal surgical microscope, as described in our previous work [22,23]. For the acquisition of FD-FLIM images, we raster-scanned a field of view of 6.5 × 6.5 mm^2^ at a working distance of 200 mm. Fluorescence spectra were acquired on smaller areas on the specimens (0.6 × 0.6 mm^2^) for the spectral range of 430 nm to 740 nm. We employed a diode laser (phoxX-405, Omicron Laserage, Rodgau, Germany) at 405 nm excitation with 6 mW of laser power at the sample plane. Post-processing routines for the fluorescence lifetime and intensity images, as well as for processing the acquired spectra, were implemented in Python (RRID:SCR_008394) as described previously [23]. For obtaining a relative measure of the PpIX peak shift, we first calculated the integral of the two bands corresponding to the two main peaks of porphyrin fluorescence (615–625 nm & 625–635 nm). We then calculated the relative contribution of the PpIX main peak (625–635 nm) with respect to the entire porphyrin spectrum. Lower numbers thereby indicate a peak shift toward the side peak at 620 nm.

(1) $$Relative\;PpIX\;peak\;contribution\;(RPPC)=\;\frac{\int_{625}^{635}Spectrum}{\int_{615}^{625}Spectrum+\int_{625}^{635}Spectrum}$$

### 2.5. Histopathological Analyses

Neuropathological tumor diagnosis was established according to the currently valid WHO classification for CNS tumors at the time of diagnosis [29,30,31]. Patients were only included if the final histopathological diagnosis confirmed the presence of a diffusely infiltrating glioma CNS WHO grades 2–4. All collected tissue samples that were analyzed with the FD-FLIM were histopathologically investigated for the presence of tumor cells. Additionally, all tissue samples were classified as tissue corresponding to tumor core, tumor infiltration zone, or non-tumor based on neuropathological analysis. 

### 2.6. Statistical Analysis

Statistical inference between tumor entities and the control group was checked with a non-parametric Mann–Whitney U test. Differences in the distributions among subgroups were ruled out with the Kolmogorov–Smirnov test after data normalization. We considered differences between groups to be significant if *p* < 0.05.

## 3. Results

### 3.1. Patient Characteristics

This study included 43 patients who underwent resection of a suspected diffuse infiltrating glioma (CNS WHO grades 2–4) after 5-ALA administration, with tissue available for FD-FLIM analysis. Of these, 12 cases showed non-significant CE, and in 31 cases, significant CE was found on preoperative MRI. The final histopathological assessment revealed a diffuse astrocytoma WHO grade 2 in seven patients, a diffuse oligodendroglioma WHO grade 2 in two patients, an anaplastic astrocytoma WHO grade 3 in four patients, and a glioblastoma WHO grade 4 was diagnosed in 30 cases. According to preoperative MRI, none of the nine WHO grade 2 tumors showed significant CE. In the group of four WHO grade 3 tumors, no significant CE on preoperative MRI was present in three cases, and significant CE was noted in one case. Finally, all 30 WHO grade 4 glioblastomas showed significant CE on MRI before surgery.

Additionally, the non-pathological tissue of three patients was included in this study and was used as a control group. During the study period, no significant side effect was noted after the oral administration of 5-ALA.

### 3.2. Glioma Resection with and Without 5-ALA Administration

WHO grade 2 gliomas: In gliomas with a final histology of a WHO grade 2, 13 samples were collected in which 52 regions of interest (ROI) were analyzed by FD-FLIM and spectroscopic measurements. None of these defined ROIs showed visible fluorescence in the group of LGG patients. Furthermore, five samples were included that were collected from two WHO grade 2 gliomas without 5-ALA administration, whereas altogether 16 ROIs were analyzed.

WHO grade 3 and 4 gliomas: In gliomas with final histology of WHO grade 3 and 4, 27 tissue samples were collected from the tumor core, and 65 fluorescing and 15 non-fluorescing ROIs were analyzed. Moreover, 18 tissue samples were derived from the tumor infiltration zone, including 18 fluorescing and 43 non-fluorescing ROIs. For the three control patients, six samples were collected, and 27 ROIs were analyzed.

### 3.3. Comparison of RPPC in Tissue with and Without Preoperative 5-ALA Administration

In the first step, we compared the mean RPPC of non-tumorous samples without 5-ALA with tissue samples of WHO grade 2 glioma samples without 5-ALA administration as well as WHO grade 2, 3, and 4 glioma samples with preoperative 5-ALA administration. Altogether, lower RPPC numbers indicate a peak shift in the fluorescence emission spectrum to the peak at 620 nm.

#### 3.3.1. Tissue Specimens Without 5-ALA Administration

**Figure 1:**
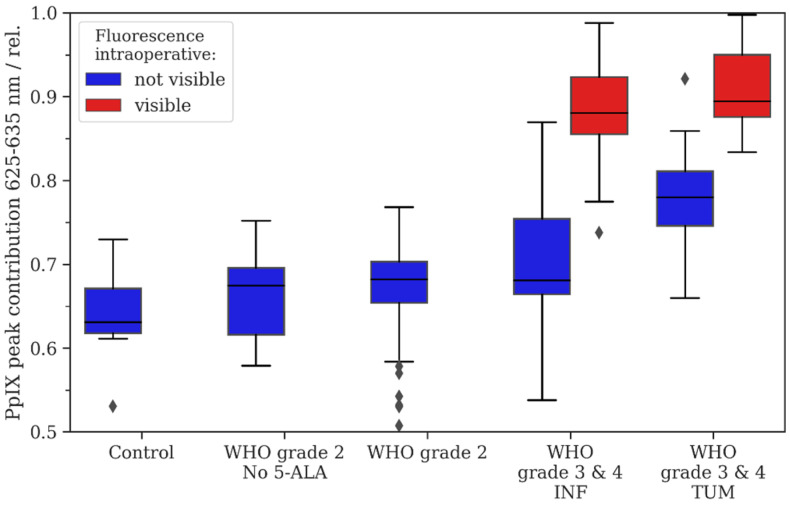
Descriptive statistics for the study cohort. The relative Protoporphyrine IX (PpIX) peak contribution was calculated as described in Equation (1), i.e., lower numbers indicate a shift in the fluorescence emission spectrum to the lower band of 615 nm–625 nm. Intraoperative fluorescence is color-coded with blue (no visible fluorescence) and red (visible fluorescence). For WHO grade 3/4 tumors, we distinguished between samples derived from tumor infiltration zone (INF) and tumor core (TUM).

The endogenous PpIX fluorescence in patients without preoperative 5-ALA administration in non-tumorous tissue had a median RPPC of 0.64, which served as a baseline for comparison of the PpIX peak shift for the following analyses. In comparison to non-tumorous tissue without 5-ALA administration, we found no significant differences in the mean RPPC compared with WHO grade 2 gliomas without 5-ALA administration (mean RPPC: 0.66; *p* = 0.193). The descriptive and inferential statistics can be found in Figure 1 and Table 1.

#### 3.3.2. Glioma Tissue After 5-ALA Administration

After preoperative 5-ALA administration, the non-fluorescing samples from WHO grade 2 gliomas (0.67) showed a significantly increased mean RPPC compared with the non-tumorous tissue without 5-ALA intake (0.64; *p* < 0.05). Furthermore, tumor core samples of WHO grade 3/4 gliomas showed a significantly increased mean RPPC (no fluorescence: 0.78; visible fluorescence: 0.91) compared with the non-tumorous tissue (no visible fluorescence: *p* < 0.05; visible fluorescence: *p* < 0.05). Additionally, samples from the tumor infiltration zone from WHO grade 3/4 tumors showed a significantly increased mean RPPC (no fluorescence: 0.71; visible fluorescence: 0.88) compared with the non-tumorous tissue (no visible fluorescence: *p* < 0.05; visible fluorescence: *p* < 0.05).

### 3.4. Analysis of RPPC in Patients with 5-ALA Administration

**Figure 2:**
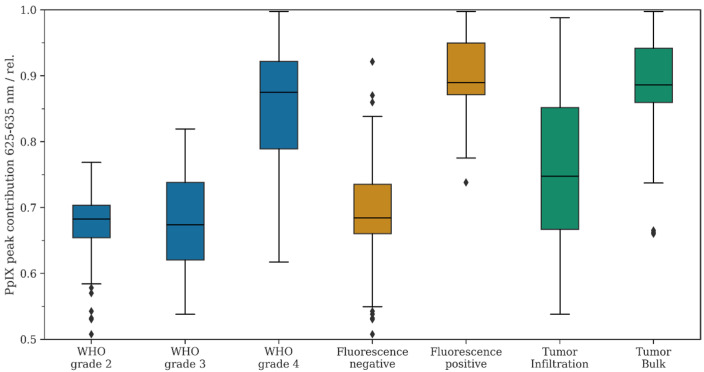
Descriptive statistics for the study cohort. The relative PpIX peak contribution was calculated as described in Equation (1), i.e., lower numbers indicate a shift in the fluorescence emission spectrum to the lower band of 615 nm–625 nm. Data from the study cohort were filtered according to WHO grade (blue), fluorescence status (orange), and tumor infiltration vs. tumor bulk (green).

In the next step, we compared the mean RPPC based on the WHO grade, fluorescence status, and tumor region in all patients with preoperative 5-ALA administration. The descriptive and inferential statistics can be found in Figure 2 and Table 2.

#### 3.4.1. Comparison of RPPC and WHO Grade

First, the mean RPPC was compared in all glioma patients with preoperative 5-ALA administration between the different WHO tumor grades. According to our data, the mean RPPC was 0.67 in WHO grade 2 gliomas, 0.70 in WHO grade 3 tumors, and 0.85 in WHO grade 4 gliomas. We observed a statistically significant difference between WHO grade 3 versus 4 gliomas (*p* < 0.05) and WHO grade 2 versus 4 gliomas (*p* < 0.05). In contrast, no significant difference was noted when comparing WHO grade 2 versus 3 gliomas (*p* = 0.37). Overall, the mean RPPC decreased in descending order of the WHO tumor grades towards the emission peak at 620 nm.

#### 3.4.2. Comparison of RPPC and Fluorescence Status

Further, we analyzed the mean RPPC for all tissue samples based on their fluorescence status (positive or negative fluorescence). The mean RPPC was 0.69 in tissue samples with no fluorescence and 0.90 in specimens with positive fluorescence. The difference in the mean RPPC between fluorescing and non-fluorescing samples was statistically significant (*p* < 0.05). Overall, we observed a decrease in the mean RPPC in non-fluorescing samples toward the second emission peak at 620 nm.

#### 3.4.3. Comparison of RPPC and Tumor Region

In gliomas with significant CE on MRI (one WHO grade 3 and 30 WHO grade 4 gliomas), we analyzed the mean RPPC for tissue samples derived from the tumor bulk and the tumor infiltration zone. According to our data, the mean RPPC was 0.89 in regions of the tumor bulk and 0.76 in regions of the infiltration zone. We found a statistically significant difference in the mean RPPC in tumor bulk samples versus samples from the tumor infiltration zone (*p* < 0.05). Overall, we observed a decrease in the mean RPPC in regions of the infiltration zone towards the second emission peak at 620 nm.

#### 3.4.4. Comparison of RPPC and Subgroup Analysis

Finally, we compared the mean RPPC in the subgroup of fluorescing/non-fluorescing tumors, as well as in fluorescing infiltrative tissue of WHO grade 3/4 gliomas with non-fluorescing WHO grade 3/4 infiltrative tissue and all non-fluorescing WHO grade 2 gliomas. According to this analysis, we found a decreased mean RPPC in the subgroup of non-fluorescing WHO grade 3/4 infiltration zone and non-fluorescing WHO grade 2 gliomas (*p* < 0.05).

### 3.5. PpIX Fluorescence Emission Peak Shift and Fluorescence Lifetime

**Figure 3:**
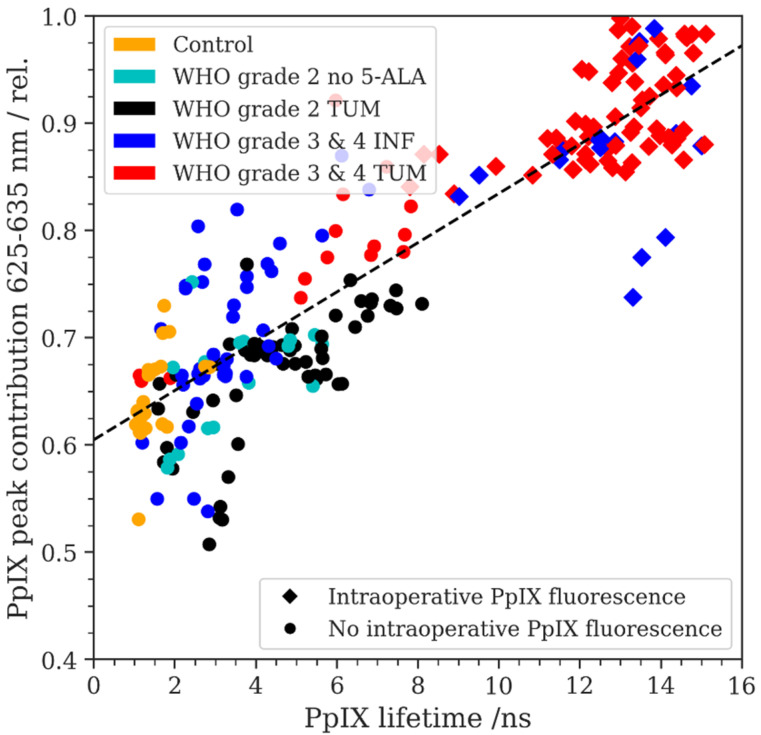
The relative PpIX peak contribution (RPPC) is plotted as a function of the PpIX fluorescence lifetime in ns, measured on the same respective ROIs. The intraoperative fluorescence status was recorded using circles (no visible fluorescence) and squares (visible fluorescence) for the data points. A linear regression fit is plotted with a dashed line (R = 0.854, R-squared = 0.729).

We then carried out a linear regression analysis for the porphyrin blue shift RPPC data and the corresponding PpIX fluorescence lifetime measurements (see Figure 3). According to our data, we found a correlation between PpIX fluorescence lifetime and RPPC (R: 0.854, R-squared: 0.729). In this sense, we found the highest fluorescence lifetimes (up to 16 ns) in the samples with a high RPPC showing visible fluorescence. In contrast, the samples with a lower RPPC from the non-tumorous group showed the lowest fluorescence lifetimes around 2 ns. Overall, fluorescence started to become visible under the microscope in the samples showing fluorescence lifetimes of approximately 8 ns and a RPPC of approximately 0.8.

## 4. Discussion

In the last few decades, different innovative and technical tools have been established to maximize the extent of resection during brain tumor surgery [32,33]. However, direct and safe intraoperative tumor visualization techniques were needed to overcome the limitations of intraoperative MRI and neuronavigation systems [32,33]. Thus, the use of fluorescent dyes was investigated in patients with different brain tumors. In addition to 5-ALA, the most common fluorescent dyes used are indocyanine green (ICG) and fluorescein sodium (Stummer et al., 2006; Acerbi et al., 2018; Cho et al., 2019a) [1,34,35]. ICG has primarily been used in vascular neurosurgery to visualize blood vessels, whereas the use of fluorescein sodium was reported in different brain tumors [34,35,36]. Both ICG and fluorescein sodium are usually injected intravenously, while 5-ALA is administered orally [4,24,35]. Currently, 5-ALA represents the most commonly used fluorescent dye in neurosurgery. In order to overcome the current limitation of poor sensitivity of 5-ALA fluorescence, new quantitative methods of fluorescence detection were developed in recent years with the aim to better distinguish non-tumorous tissue from tumor tissue by detecting “subvisual” fluorescence that is not visible with conventional 5-ALA technology [17,18,22,37]. One promising method represents the spectroscopic analysis of PpIX accumulation for the quantitative assessment of intratumoral fluorescence [17,18,27,38]. Another powerful technique applied at our center is FD-FLIM, which is capable of quantifying the fluorescence lifetime in brain tumor tissue and can also be combined with spectroscopic measurements [22,23].

Generally, the emission spectrum of PpIX after oral administration of 5-ALA is located at 635 nm wavelength [24]. In recent years, Montcel et al. performed a spectroscopic analysis of 35 tissue samples from four patients (two LGG, two HGG), including different regions such as normal tissue, tumor infiltration zone, and solid tumor [39]. Interestingly, the authors observed a relevant PpIX emission peak shift towards 620 nm in regions of the tumor infiltration zone of HGG as well as LGG tissue in their first analyses. According to the data, this emission peak at 620 nm in these specific tumor areas seemed to be the main contributor to the overall fluorescence signal [39]. In this sense, the authors were able to distinguish solid tumor areas of HGG from LGG and tumor infiltration zones of HGG [39]. In a further study by this group, the two emission spectra of PpIX were investigated in 10 patients (6 HGG, 4 LGG) [26]. Similarly, this study found that the main contribution of the PpIX fluorescence peak is localized at 635 nm in HGG and in margins with high cell density [26]. In contrast, LGG and low cell density margins of HGG showed the main contribution of PpIX at the second peak, at 620 nm [26]. Therefore, Montcel et al. suggested that the second emission peak of PpIX at 620 nm should be considered in the analysis of 5-ALA-induced PpIX accumulation [40]. Although the studies of Montcel et al. demonstrated remarkable observations, these first studies were limited by their low patient number [26,40]. In a previous study using an intraoperative spectroscopic probe, we observed a shifted peak at 627 nm aside from the main PpIX peak at 635 nm. We suspected that this additional peak is especially present in tissues with very low PpIX concentrations [27]. A retrospective ex vivo analysis by Molina et al., including close to 600 specimens of 130 patients, shed light on the composition of the fluorescence at 620 nm and suggested that other porphyrins, such as coproporphyrin III, contribute significantly to the signal with less contribution of the second photo-state of PpIX [28]. Our present study included altogether 46 patients with glioma resection of different WHO grades (grades 2–4) to investigate the shift toward the second emission peak at 620 nm and to analyze the implications for the fluorescence lifetime in brain tumor surgery. For this purpose, we analyzed different glioma samples after preoperative 5-ALA administration and without preoperative 5-ALA intake in our cohort using FD-FLIM and spectroscopic measurements.

After preoperative 5-ALA administration, all samples from WHO grades 2–4 gliomas showed a significantly increased mean RPPC compared with non-tumorous tissue without 5-ALA intake. Thus, these findings confirm that preoperative 5-ALA administration increases the mean RPPC in glioma tissue from the second emission peak at 620 nm towards the main emission peak at 635 nm. In the next step, we compared the mean RPPC based on the WHO grade, fluorescence status, and tumor region in all patients with preoperative 5-ALA administration. According to these data, the mean RPPC showed a decrease in descending order of the WHO tumor grades towards the second PpIX emission peak at 620 nm. Further, we observed a decrease in the mean RPPC in non-fluorescing samples as well as in regions of the infiltration zone towards the second emission peak at 620 nm as compared with fluorescing and solid tumor regions. In a further analysis, we compared the mean RPPC in fluorescing/non-fluorescing tumor WHO grade 3/4 glioma samples as well as fluorescing infiltrative tissue of WHO grade 3/4 gliomas with non-fluorescing WHO grade 3/4 infiltrative tissue and all non-fluorescing WHO grade 2 gliomas. According to this data, we found a decreased mean RPPC in the subgroup of non-fluorescing WHO grade 3/4 infiltration zone and non-fluorescing WHO grade 2 gliomas. Therefore, we found a shift from the main emission peak at 635 nm towards the second emission peak at 620 nm in non-fluorescing infiltrative tissue of WHO grade 3/4 gliomas and WHO grade 2 gliomas. Altogether, our data are in line with previous studies, leading to the assumption that the emission peak at 620 nm and porphyrin fluorescence represent a large contribution in LGG and tumor infiltration zones [27,28,39,40]. To our knowledge, this represents the first systematic and detailed analysis of the peak shift effect on FLIM measurements in a large cohort of different WHO glioma grades, including samples with different visible fluorescence status.

We additionally correlated the RPPC with the corresponding PpIX fluorescence lifetime in each tumor subgroup. A peak shift in the porphyrin spectrum was correlated with reduced fluorescence lifetimes. Since the measured fluorescence lifetime is a mixture of both the autofluorescence base spectra (flavin, lipofuscin, NADH) as well as various porphyrins, including PpIX, understanding the average fluorescence lifetime and its implications for diagnostics requires an in-depth understanding of the contributing fluorophores. Differences in the main components of the two emission peaks in tumors might be based on a different aggregation of PpIX produced by LGG and the margins of HGG based on different tumor microenvironments [26,39]. It was also shown that the distribution of PpIX in healthy tissue is mainly localized in mitochondria, whereas, in tumor tissue, PpIX can be found extramitochondrial and in the cytosol [41]. These findings might explain the existence of different fluorescence lifetimes and PpIX fluorescence based on the tumor microenvironment. In detail, this study showed that PpIX has a lifetime of 16.4 ns in organic solutions, compared with 5.4–7.5 ns in mitochondria and 2–4 ns in the cytoplasm [41]. In particular, for lower concentrations of PpIX, the overall fluorescence lifetime measured is increasingly influenced by the fluorescence lifetimes of endogenous fluorophores, emitting in the same spectral range as PpIX [42]. This adds up to the variances measured for PpIX lifetimes in different tumor microenvironments. Furthermore, the fluorescence by other porphyrins, such as coproporphyrin III, adds to the complexity. Previous studies showed two main components of the lifetime of coproporphyrin (2.8 ns bound form, 15 ns free form) depending on its environment and interaction with other molecules [43]. Further research to understand the peak shift in porphyrin fluorescence is crucial and will contribute to a better understanding of the fluorescence lifetimes in brain tumor tissue for intraoperative fluorescence-based diagnostics.

A limitation of our study is that the measurements were performed ex vivo within one hour of tissue collection in a laboratory. Therefore, the results may not be fully comparable to an in vivo scenario. Moreover, in this study, the patients’ histopathological diagnoses were established according to the valid WHO tumor classification at the time of surgery [30,31]. Comparing patients with diagnoses stated through different WHO classification systems has to be mentioned as a limitation of this study. Furthermore, our analysis was based on a linear decoupling of the fluorescence background rather than a spectral unmixing approach, which requires knowledge of the baseline spectra and is the best method to analyze these signals [44]. Nevertheless, the linear decoupling of autofluorescence in this study allowed us to look for significant differences within groups, as this potential systematic error is balanced across different spectra. The RPPC was defined as a quantitative measure to compare between groups and then correlate the blue shift with the respective fluorescence lifetime. In addition, spectral unmixing methods are complicated by inhomogeneities in the tissue due to different concentrations of the basic fluorophores and differences between the various tumor types.

The knowledge about the second emission peak at 620 nm, especially in LGG and the non-fluorescing infiltration zone of WHO grade 3/4 gliomas, is crucial. By including the emission peak at 620 nm in PpIX analysis, tumor tissue might be better distinguished from healthy tissue, especially in LGG tissue and the non-fluorescing infiltration zone of WHO grade 3/4 gliomas [26,39,40]. This approach should be investigated during intraoperative spectroscopic and FD-FLIM PpIX analysis, with the aim of maximizing the extent of safe resection in these specific tumor regions, thus improving patient prognosis. Future studies using quantitative fluorescence techniques are warranted to further investigate the relevance of the two PpIX emission peaks in different intratumoral areas. Based on these results, quantitative and visual 5-ALA fluorescence techniques might be improved to visualize fluorescence more sensitively.

## 5. Conclusions

In this study, we analyzed the presence of a two-peaked emission spectrum of PpIX fluorescence in varying intratumoral areas and regions with different visible fluorescence status in WHO grades 2–4 gliomas based on a quantification of the relative PpIX peak shift and showed the influence on the fluorescence lifetime. According to our data, the emission peak shift decreases towards the second peak at 620 nm in descending order of WHO grades, non-fluorescing samples, and infiltrative tumor regions. These findings highlight that the second emission peak at 620 nm plays a non-negligible role in LGG and the non-fluorescing infiltration zone. To overcome the current limitation of 5-ALA fluorescence in sensitivity, we must be aware of the two-peaked emission spectrum in gliomas, which markedly impacts quantitative measurements as the fluorescence lifetime. Knowledge about the main contribution of the PpIX emission peak in LGG and the tumor infiltration zone at 620 nm will improve intraoperative tumor visualization using 5-ALA fluorescence in glioma surgery in the future.

## Figures and Tables

**Table 1:** Descriptive and inferential data for the relative PpIX peak contribution as described in Equation (1) and visualized in Figure 1. Statistical inference was tested for all groups with respect to the control group.

	Control	WHO Grade 2 No 5-ALA	WHO Grade 2 with 5-ALA	WHO Grade 3/4 INF		WHO Grade 3/4 TUM	
Intraoperative fluorescence-status	not visible	not visible	not visible	not visible	visible	not visible	visible
N samples	6	5	13	18		27	
N regions of interest (ROI)	27	16	52	43	18	15	65
Mean	0.64	0.66	0.67	0.71	0.88	0.78	0.91
Median	0.63	0.68	0.68	0.68	0.88	0.78	0.89
0.25 quartile	0.62	0.62	0.65	0.66	0.86	0.75	0.88
0.75 quartile	0.67	0.7	0.7	0.75	0.92	0.81	0.95
*p*-value	-	*p* = 0.193	*p* = 0.007	*p* = 0.001	*p* = < 0.001	*p* = < 0.001	*p* = < 0.001

**Table 2:** Descriptive and inferential data for the relative PpIX peak contribution as described in Equation (1) and visualized in Figure 2.

	WHO Grade 2	WHO Grade 3	WHO Grade 4	Fluorescence Negative	Fluorescence Positive	Tumor Infiltration	Tumor Bulk
N ROI	52	19	122	126	83	61	80
Mean	0.67	0.70	0.85	0.69	0.90	0.76	0.89
Median	0.68	0.67	0.88	0.68	0.89	0.75	0.89
0.25 quartile	0.65	0.62	0.79	0.66	0.87	0.67	0.86
0.75 quartile	0.70	0.74	0.92	0.73	0.95	0.85	0.94
*p*-value	WHO grade 2 vs. 4: *p* = < 0.001	WHO grade 3 vs. grade 2: *p* = 0.365	WHO grade 4 vs. 3: *p* = < 0.001	-	Pos. vs. neg. *p* = < 0.001	-	Bulk vs. Inf. *p* = < 0.001

ROI: regions of interest.

## Data Availability

Data supporting reported results can be requested by contacting the corresponding author.
